# Nanoindentation of Chromium Oxide Possessing Superior Hardness among Atomic-Layer-Deposited Oxides

**DOI:** 10.3390/nano12010082

**Published:** 2021-12-29

**Authors:** Taivo Jõgiaas, Aivar Tarre, Hugo Mändar, Jekaterina Kozlova, Aile Tamm

**Affiliations:** Department of Materials Science, Institute of Physics, University of Tartu, W. Ostwald Str. 1, 50411 Tartu, Estonia; aivar.tarre@ut.ee (A.T.); hugo.mandar@ut.ee (H.M.); jekaterina.kozlova@ut.ee (J.K.); aile.tamm@ut.ee (A.T.)

**Keywords:** nanoindentation, chromium (III) oxide, atomic layer deposition, hardness, elastic modulus

## Abstract

Chromium (III) oxide is a technologically interesting material with attractive chemical, catalytic, magnetic and mechanical properties. It can be produced by different chemical and physical methods, for instance, by metal–organic chemical vapor deposition, thermal decomposition of chromium nitrate Cr(NO_3_)_3_ or ammonium dichromate (NH_4_)_2_Cr_2_O_7_, magnetron sputtering and atomic layer deposition. The latter method was used in the current work to deposit Cr_2_O_3_ thin films with thicknesses from 28 to 400 nm at deposition temperatures from 330 to 465 °C. The phase composition, crystallite size, hardness and modulus of elasticity were measured. The deposited Cr_2_O_3_ thin films had different structures from X-ray amorphous to crystalline α-Cr_2_O_3_ (eskolaite) structures. The averaged hardness of the films on SiO_2_ glass substrate varied from 12 to 22 GPa and the moduli were in the range of 76–180 GPa, as determined by nanoindentation. Lower values included some influence from a softer deposition substrate. The results indicate that Cr_2_O_3_ could be a promising material as a mechanically protective thin film applicable, for instance, in micro-electromechanical devices.

## 1. Introduction

Chromium (III) oxide has been researched as a corrosion- and wear-resistant, mechanically protective hard coating, a component in glasses and glass ceramics, a cermet-type hard material or in other composites or as a sintering additive [1,2,3,4,5,6,7,8,9,10,11,12]. Some research has been focused on doped and pristine Cr_2_O_3_’s optical, magnetic, catalytic or sensory properties [13,14,15,16,17,18,19,20,21,22,23].

A number of different physical and chemical methods have been employed to produce Cr_2_O_3_ or the materials containing it. The physical techniques have included detonation and plasma spraying, different magnetron sputtering techniques and pulsed laser deposition [1,2,10,11,12,15,24]. The chemical techniques have included thermal decomposition of or co-precipitation from Cr(NO_3_)_3_∙9H_2_O, chemical vapor deposition (CVD) from CrCl_3_ and H_2_O at 900 °C or CrO_3_ and I_2_ at 500 °C, metal–organic chemical vapor deposition from Cr(CO)_6_, laser-assisted CVD from O_2_ and Cr(CO)_6_ and atomic layer deposition (ALD) from CrO_2_Cl_2_ and CH_3_OH precursors or from chromium acetyl acetonate and ozone [9,13,14,25,26,27,28,29].

Aside from the usual thin film deposition, some of the used techniques have resulted in various appearances of Cr_2_O_3_. For instance, the CVD from CrCl_3_ and H_2_O at 900 °C resulted in single crystalline Cr_2_O_3_ nanowires and nanobelts [14]. The decomposition of Cr(NO_3_)_3_ ∙ 9H_2_O, mixed with zirconia–toughened alumina at 600 °C, gave microparticles, whereas the co-precipitation from the same precursor resulted in nanoparticles [9,13]. CVD using CrO_3_ and I_2_ at 500 °C as precursors resulted in Cr_2_O_3_ flakes [21].

The previous reports have indicated the Cr_2_O_3_ thin films possess a high hardness of over 20 GPa [10,14,15,30,31,32]. In some conditions the processes produced chromia with lower hardness, even down to an 8–10 GPa level [14]. Reported values for the moduli range from 160 to about 290 GPa. The lower hardness and modulus values tend to appear in the amorphous chromia, but the crystalline counterparts have higher values [10,14,15]. It has been shown that Cr_2_O_3_, alloyed with other elements, could result in a super-hard material [10].

Compared to previously noted techniques, ALD allows to deposit conformal thin films on basically arbitrary three-dimensional shapes through the employment of the self-limiting irreversible and saturated surface reactions [33]. This feature makes ALD a very good tool to coat parts of micro-electromechanical devices or other systems to create surfaces with modified mechanical properties or, for instance, to act as a catalyst on a mesoporous substrate [34,35,36,37,38].

Even though Cr_2_O_3_ has been deposited before using ALD, the mechanical properties have not been reported [21,22]. The present work was dedicated to the investigation of the viability of atomic-layer-deposited Cr_2_O_3_ as a potentially hard and stiff material to be used as a mechanically protective or modifying thin film coating in micro- or nanoelectromechanical devices [31]. The crystallinity, the effect of the growth temperature and the thickness of the thin films were correlated to the nanoindentation hardness and modulus.

## 2. Experimental

The Cr_2_O_3_ thin films were deposited from CrO_2_Cl_2_ and CH_3_OH (Sigma-Aldrich, Darmstadt, Germany) in an in-house built atomic layer deposition reactor [39]. The detailed description and results can be found in previously published article [26]. Briefly, the films were grown on fused silica substrates at the growth temperature T_g_ of 330, 375, 420 and 465 °C. The cycle count was varied between 400–4300 ALD cycles. Each cycle consisted of 0.2 s exposure to CrO_2_Cl_2_, 2 s purge with dry N_2_, 2 s exposure to CH_3_OH vapors and another 2 s N_2_ purge. Both precursors were volatilized at −20 °C.

The crystallographic phase composition and the apparent crystallite size D_v_ were determined in direction of the normal to the lattice plane (104) by using Voigt decomposition analysis and SRM-660 (NIST, Gaithersburg, MD, USA) as reference material for diffraction peak instrumental broadening calibration [40]. Thickness of the films was determined using X-ray diffraction (XRD) and reflectivity (XRR) methods on diffractometer SmartLab™ (Rigaku, Tokyo, Japan) working at 8.1kW (Cu Kα radiation, λ = 0.154178 nm). Asymmetric 2θ-scan at fixed grazing incidence angle of ω = 0.42° (GIXRD) was used for phase composition analysis. In the GIXRD experiment, the D_v_ was determined in direction that is approximately 16.6° inclined to the sample surface normal. Texture coefficient of reflections (T_hkl_) was calculated from the following equation [41]: T_hkl_ = I^OBS^_hkl_/I^DB^_hkl_/<I^OBS^>, where I^OBS^_hkl_ is the observed absolute intensity of the reflection hkl; I^DB^_hkl_ is the relative intensity of reference reflection hkl in the database; and <I^OBS^> is an average over all observed reflections of the ratio of observed to reference peak intensity values. T_hkl_ is close to 1.0 for a randomly oriented polycrystalline sample and approaches to the number of observed reflections for a sample showing high preferred (hkl) orientation.

High-resolution scanning transmission electron microscopy (STEM) investigations were carried out by using a Titan Themis 200 microscope (FEI, Hillsboro, OR, USA) equipped with a Cs-probe corrector and operated at 200 kV. A sample for the STEM study in the form of thin cross-sectional lamella was prepared by a focused ion beam by using an in-situ lift-off technique in the scanning electron microscope Helios NanoLab 600 Dual Beam system (FEI, Hillsboro, OR, USA). Prior to the lamella preparation procedure, a platinum layer was deposited on the specimen by magnetron sputtering to minimize the charging of the specimen and to protect the chromium oxide film from the ion beam milling effect.

The hardness and elastic modulus of ALD Cr_2_O_3_ films were investigated by using nanoindentation device Bruker Hysitron Triboindenter TI 980 (Billerica, MA, USA; Minneapolis, MN, USA). The samples were measured in continuous stiffness mode by using a Berkovich-type diamond tip. The device was calibrated by using a fused quartz standard (Bruker) with defined hardness of 9.25 GPa and reduced modulus of 69.6 GPa. The overall arithmetic average of 17 measurements of the fused quartz was 9.18 GPa for hardness and 70.1 GPa for modulus. The strain rate of 0.05 nm/s and tip vibration frequency (220 Hz) were kept constant during the calibration and measurements. The calibrations were performed before and after measurements. The latter results indicated that the tip was still in good condition and assured the correctness of the measurements. The calibrations indicated that the valid displacement range was approximately from 20 nm to 100 nm. In the given range, the deposition substrate (SiO_2_ glass) had hardness of 8 ± 0.5 GPa and modulus of 61 ± 1.5 GPa (here, the error margins indicate the absolute minimum and maximum deviation at a given measurement point). The standard deviations of a single measurement remained near 3 GPa for modulus and 0.3 GPa for hardness at displacements around 20 nm. The deviations somewhat reduced towards higher displacements. Any single indentation measurement consisted of 61 data points in total, spread along the displacement range. The data points below displacements of 15 nm were removed. The data points near 20 nm of displacement were considered through analysis.

Due to the fact that some of the thin films were very thin in this study, the fused SiO_2_ glass made a good deposition substrate as it was free of any pile-up, sink-in or cracking effects which might occur during nanoindentation, possibly discrediting data analysis. Additionally, this is the reason for SiO_2_ glass usage as a calibration standard. As a glassy material, the substrate should have isotropic mechanical properties, which should avoid problems, such as film-induced cracking of a substrate [42].

Thirty separate continuous stiffness measurements were performed on each sample. Some low-quality measurements were removed, for instance, because of unsuitably high or unstable (thermal or positioning) drift rate of 0.29 nm/s, reducing the count of acceptable measurements by 5 in the worst case. Maximum drift level of 0.05 nm/s was the default acceptance level.

## 3. Results and Discussion

### 3.1. X-ray Diffraction

The crystallinity affects the (mechanical) properties of a material. Therefore, it is essential to measure the deposited thin film crystallographic properties, such as crystallinity and crystallite size.

**Figure 1:**
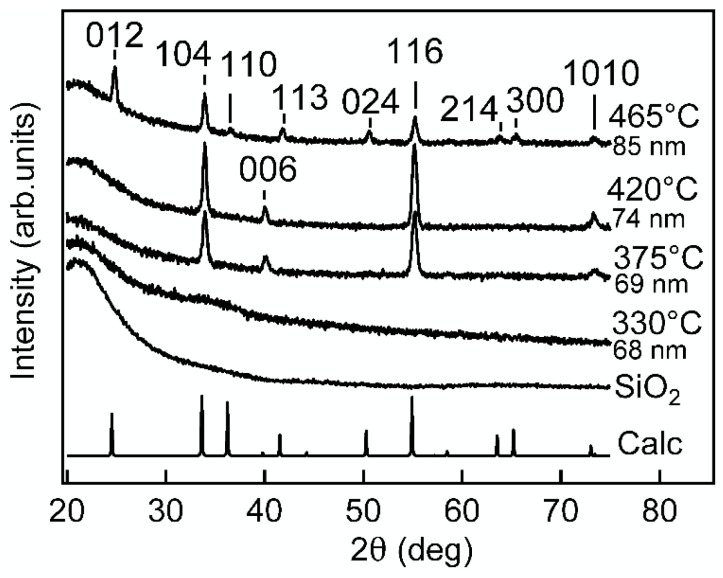
Diffraction patterns of ALD Cr_2_O_3_ films grown at different temperatures. The thin film thicknesses were 68–85 nm. The diffraction pattern denoted as Calc was calculated from crystal structure data of eskolaite corresponding to the inorganic crystal structure database collection code 261801. The labels at the reflections are the Miller indices of corresponding lattice planes.

Figure 1 shows that the crystallinity increased with the increase of the growth temperature T_g_. The sample deposited at 330 °C was X-ray amorphous, but the samples deposited at higher temperatures showed reflections that were identified as belonging to α-Cr_2_O_3_ (mineral eskolaite, ICDD PDF-2 file number 01-078-5435).

**Figure 2:**
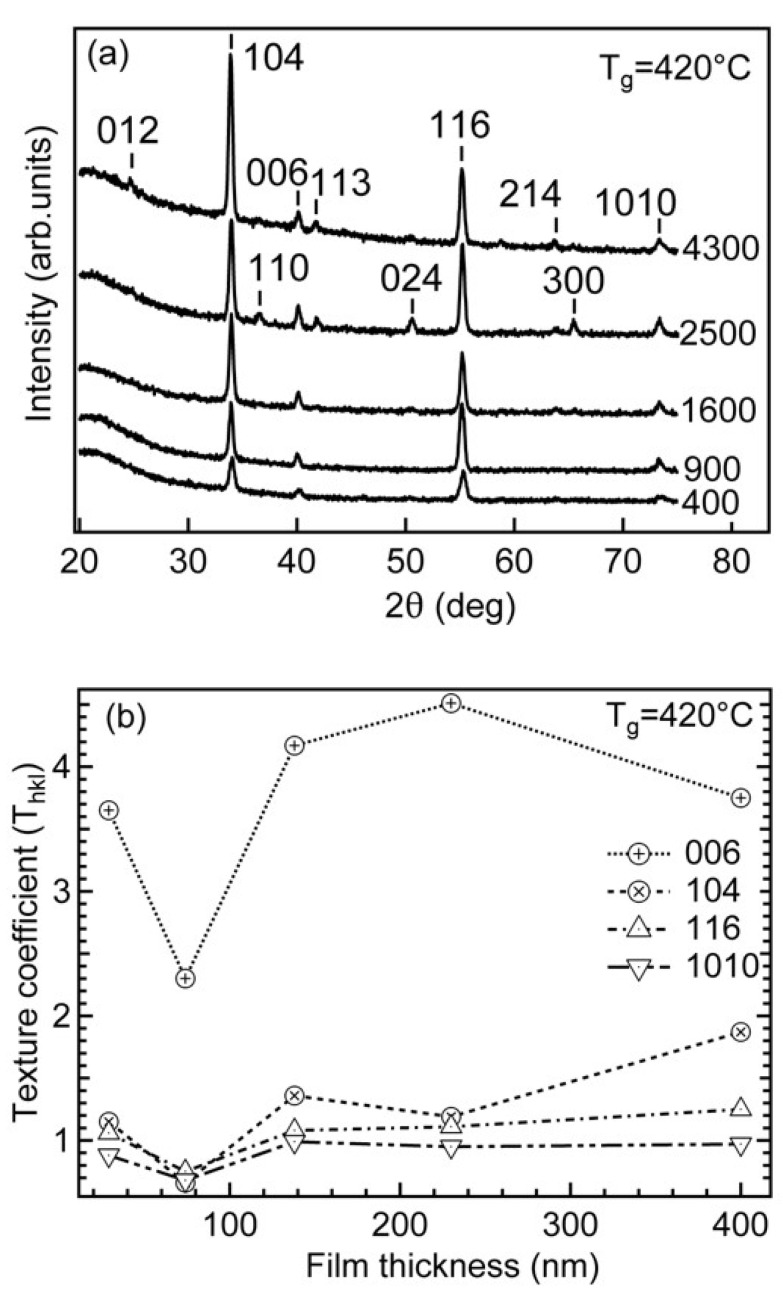
Diffraction patterns of chromia thin films with different cycle counts (**a**,**b**) variation of texture coefficient T_hkl_ for the dominating reflections (104), (006), (116) and (1010) as a function of the film thickness (**b**) deposited at 420 °C.

The diffractograms of thin films with different cycle counts are shown in Figure 2.

Figure 2b shows the variation of texture coefficient T_hkl_ for the dominating reflections (104), (006), (116) and (1010) as a function of the film thickness. The value of T_hkl_ for the reflection 006 was a few times higher compared to the values for the other reflections. Pang et al. showed that the mechanical properties of Cr_2_O_3_ films change with the texture [30]. For instance, a film with strong (300) texture had hardness of about 28 GPa but was reduced to 20 GPa for a low-textured film. Similar results were gained by Luo et al. [15].

Considering that, in the GIXRD analysis, these observed reflections were generated from lattice planes that were inclined to the sample surface in the angle range of 20–38° and, by assuming modest mosaicity of the film, it can be concluded that the preferred growth direction of the films was close to the crystallographic [001] direction. The mosaicity of the films was determined and the preferred growth plane characterized in more detail also by symmetrical θ/2θ and in-plane XRD analysis for the two thickest samples (230 and 400 nm) that allowed to record diffraction reflections with acceptable signal-to-noise ratio for both of the analysis modes.

**Figure 3:**
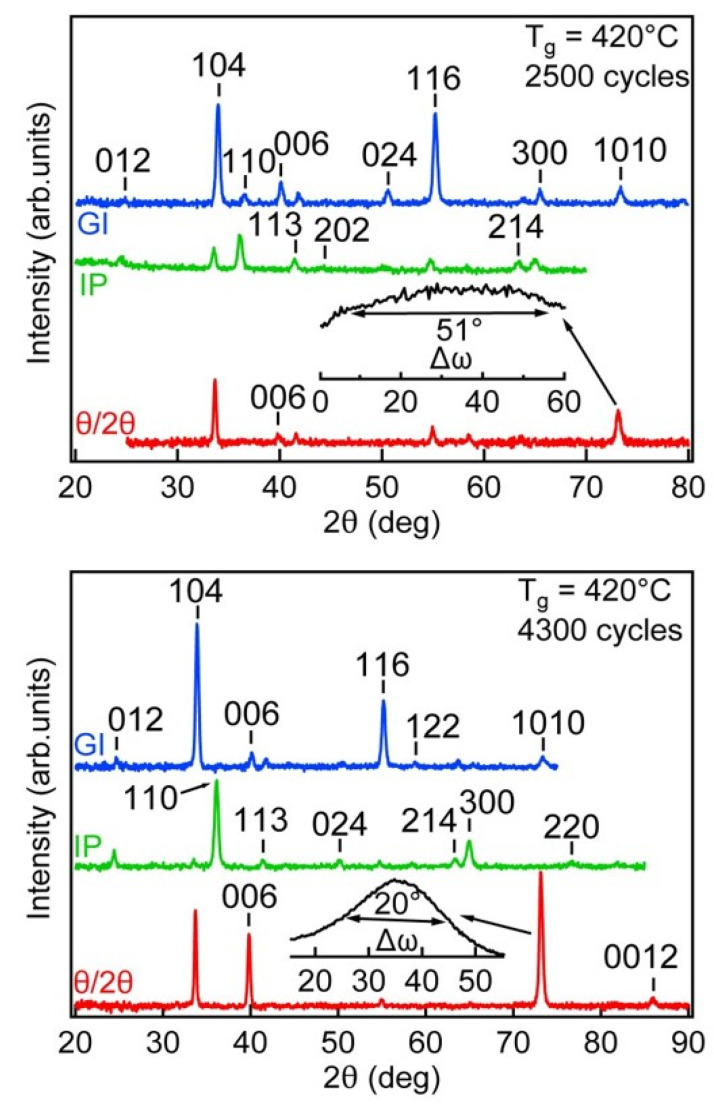
XRD patterns recorded in grazing incidence (GI), in-plane (IP) and symmetrical (Θ/2Θ) setup for film grown at 420 °C with 2500 and 4300 cycles. The inset above the Θ/2Θ -XRD pattern presents rocking curve (*ω*-scan) of 1010 reflection showing FWHM of approximately 51° for 2500 cycles and 20° for 4300 cycles.

The θ/2θ-XRD pattern of the film deposited with 2500 cycles showed (bottom curve in Figure 3) two strong reflections (104 and 1010) and four weak reflections (006, 113, 116, 122), whereas the IP-XRD pattern exhibited two stronger reflections from the (104) and (110) lattice planes and weaker reflections from the (113), (202), (116), (214) and (300) planes. The appearance of the θ/2θ-reflection from the plane (104) and IP reflection from plane (110) are a clear indication that the preferred growth of this film was in the (104) plane or in the plane where the inclination angle relative to (104) plane was small. The high value of the full width of the half maximum (FWHM) of the 1010 reflection (∆*ω* = 51°) suggests that the mosaicity of this film was very high and the preferred growth direction varied approximately from −25 to +25° relative to the surface normal of the (104) plane. The refinement of the cell parameters determined from the IP and θ/2θ-XRD patterns and calculation of the lattice strain, relative to the parameters from the database (ICDD PDF-2), showed that the crystal lattice was slightly compressed (0.05–0.1%) in the direction of the surface normal and stretched approximately parallel to the surface by the same amount.

The IP and θ/2θ-XRD patterns from the film deposited with 4300 cycles showed (Figure 3) that, beside the (104) plane, the (001) plane appeared as a preferred growth plane. A relatively high intensity of the 006 reflection, the appearance of the 0012 reflection on the θ/2θ-XRD pattern, the two times smaller FWHM of the rocking curve of the 1010 reflection and the approximately 2.5–3 times larger values of the lattice strain (compared to the film deposited with 2500 cycles), but also strong 110 and 300 reflections on the IP-XRD patterns of this film, allowed us to conclude that, in thicker films (above 230 nm), the mosaicity decrease is accompanied by a preferred growth in the (001) plane. 

**Figure 4:**
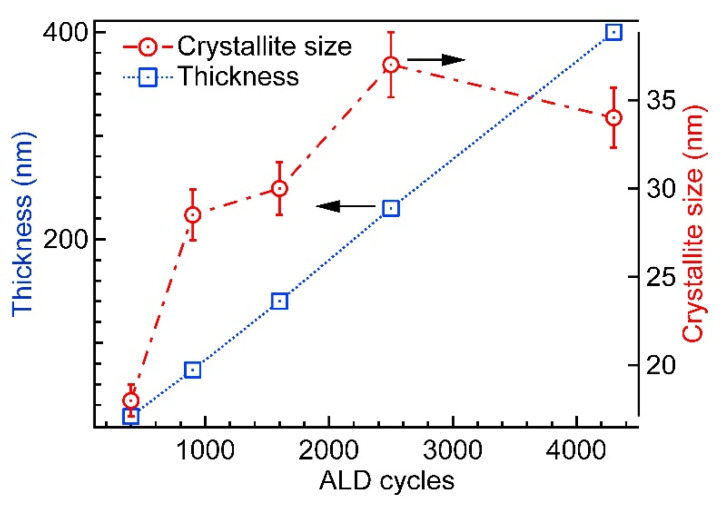
Variation of film thickness and XRD apparent crystallite size (D_y_) with the number of deposition cycles for Cr_2_O_3_ samples grown at 420 °C.

The results of the film thicknesses deposited at 420 °C and the respective crystallite sizes are presented in Figure 4. The thickness measurement error for the thickest film was around 10 nm and for thinner films (below 100 nm) from 1 to 3 nm, reducing in coherence with the film thickness. 

The growth rate of Cr_2_O_3_ at 420 °C, calculated from the slope of the film thickness in variation with ALD cycles, was 0.096 nm/cycle (determination coefficient R^2^ = 0.99). The linear correlation of the cycle count and resultant thin film thickness are characteristic of ALD.

It can be seen that the apparent crystallite size (D_v_) depended remarkably on the number of growth cycles or, in other words, on film thickness. The crystallites grew fast during the first 900 cycles of deposition, reaching the size of about 30 nm. The further increase of crystallites proceeded considerably slower—within the next 1300 cycles the increase was only about 7 nm. 

**Figure 5:**
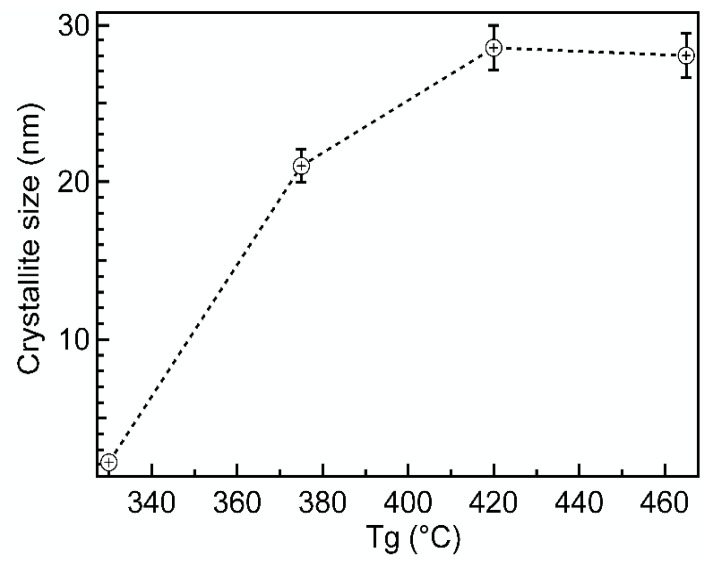
X-ray apparent crystallite size (D_v_) in variation of deposition temperature (T_g_) for Cr_2_O_3_ films. The thickness of the films was in the range of 68–85 nm.

The deposition temperature T_g_ was also an important factor that controlled the crystallite size of Cr_2_O_3_. Figure 5 shows that D_v_ increased fast with the increase of T_g_ from 330 to 375 °C. Starting from T_g_ = 380 °C, the increase slowed down and saturated finally at 420 °C, reaching the maximum value of about 27–30 nm. 

Khojieri et al. deposited at room temperature approximately 80 nm thick films of Cr_2_O_3_ on monocrystalline silicon by using DC magnetron sputtering [29]. Annealing a sample at 400 °C resulted in grain sizes approximately 28–31 nm, which is comparable to current work results.

The deposition conditions and the respective apparent crystallite size, film thickness and other results, determined by X-ray measurements, are gathered in Table 1. The integral width β of a reflection was the parameter that was used for calculation of crystallite size. Here it is presented for (104) reflection. Respective values can be seen in Table 1, which correspond to apparent X-ray crystallite size of 20–30 nm. The unit cell parameters compare well to database values.

### 3.2. Nanoindentation

#### 3.2.1. Relation of Hardness and Modulus to the Deposition Temperature

**Figure 6:**
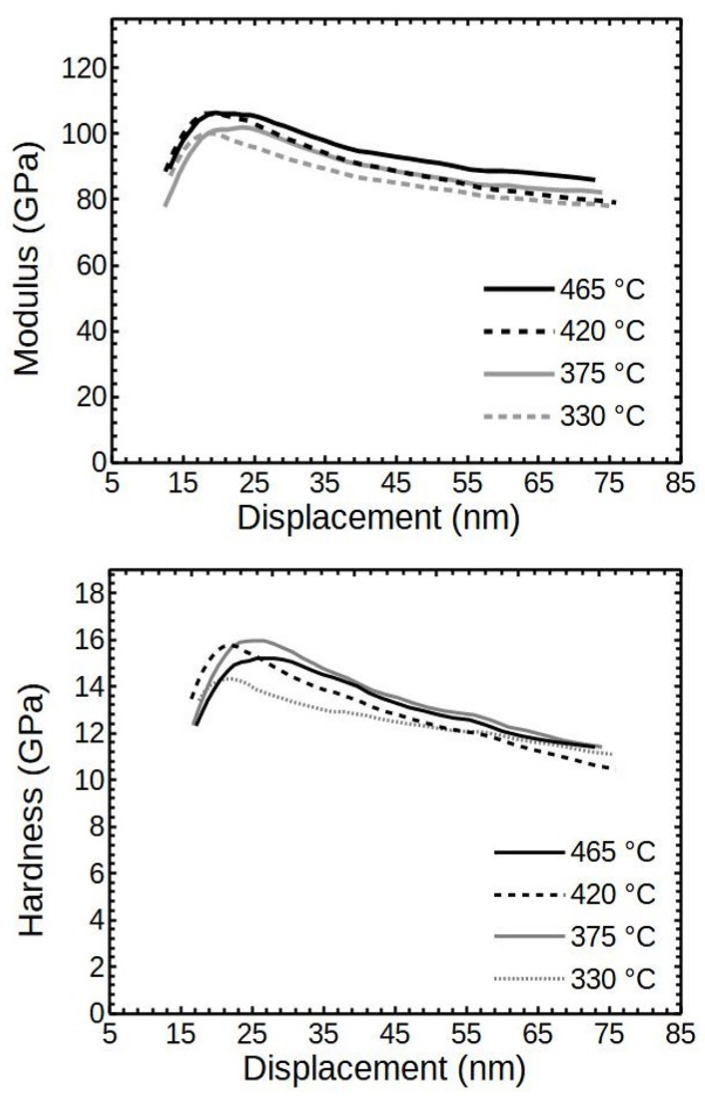
The averaged modulus and hardness values of ALD Cr_2_O_3_ deposited at different temperatures. Cr_2_O_3_ layer thicknesses are around 68–85 nm.

The relation of the deposition temperature T_g_ and the averaged reduced moduli of the films with thicknesses of 68–85 nm is shown in Figure 6. It can be seen that there was some dependence of moduli on T_g_, but the difference was low—about 10 GPa (~10%) in between X-ray amorphous and crystalline thin films at the displacement of 20 nm. A likely reason for such low differences was the influence of a softer SiO_2_ substrate. Considering that the thin films had thicknesses of only about 68–85 nm, at the displacements of 20 nm, the substrate reduced the measured values and the differences thereof.

The differences in the averaged hardness values were within 15% (Figure 6). The higher values corresponded to the films with the appearance of the crystalline Cr_2_O_3_. 

Pang et al. showed that the hardness and modulus of chromia thin film do increase with the increase in crystallinity [28]. They used reactive magnetron sputtering to produce Cr_2_O_3_ films and measured the range of hardness to be 11–21 GPa and 170–234 GPa for modulus, depending on the crystallinity. Khojier et al. measured the hardness of DC magnetron sputtered and annealed at 300 °C and 400 °C samples to be around 17–21 GPa and the respective moduli around 160–185 GPa [29].

**Figure 7:**
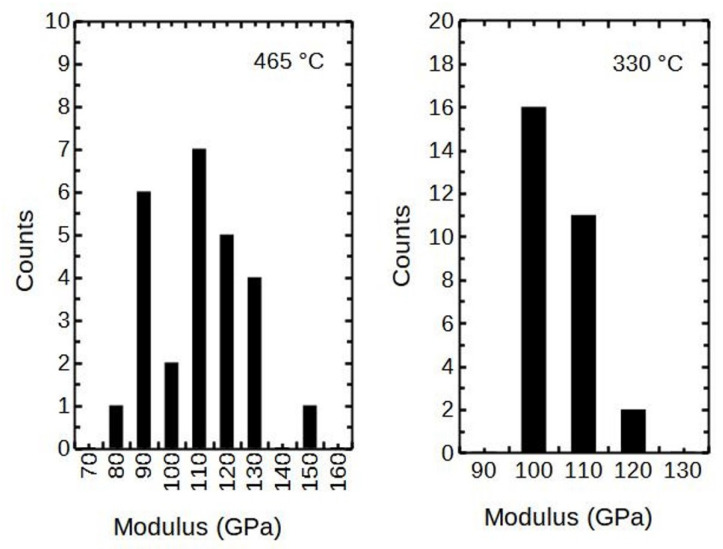
The scatter of modulus measurements of the 85 and 68 nm thick films, respectively.

The scattering of the nanoindentation results taken at 20 nm of displacement are presented in the histograms (Figure 7). It can be seen that the X-ray amorphous thin film deposited at 330 °C had lower scattering (±10 GPa) than the crystalline counterpart (±35 GPa) deposited at 465 °C. As an overall notice, the scatter widened with the growth temperature T_g_. 

#### 3.2.2. Relation between Hardness, Modulus and Thickness of Chromia

**Figure 8:**
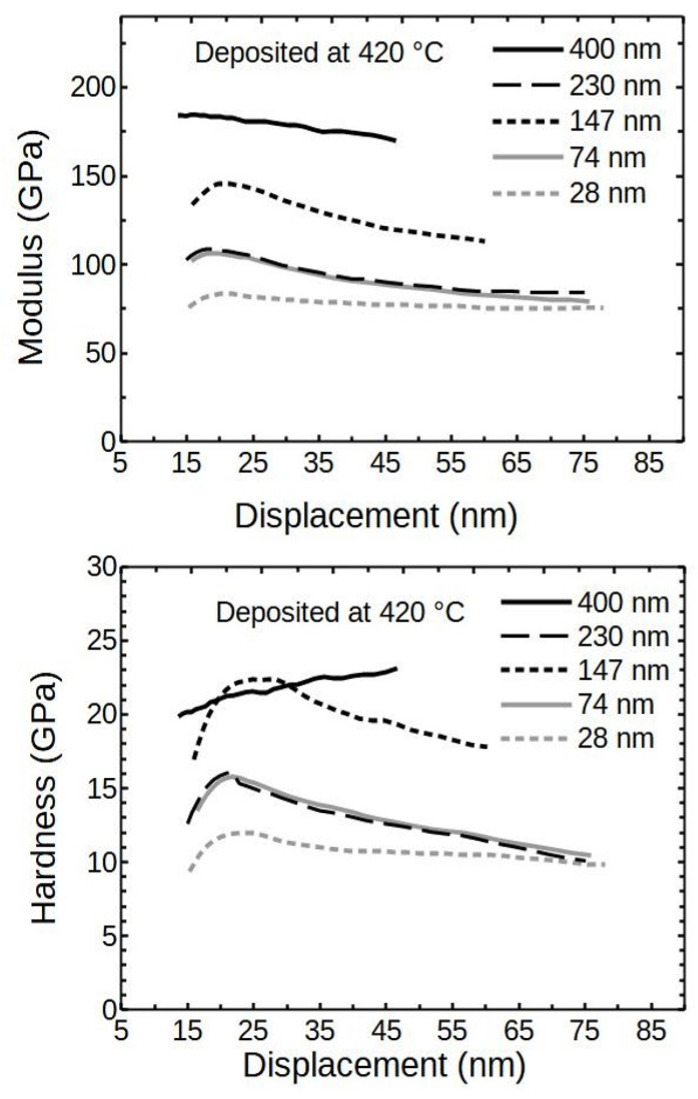
Modulus and hardness of ALD Cr_2_O_3_ thin films deposited at 420 °C.

Figure 8 visualizes the relation between the modulus and the deposited thin film thickness (deposited at 420 °C). It indicates that the softer substrate has a reducing impact on the results, which diminished inversely with film thickness. The hardness showed similar trends as the moduli (Figure 8). 

It can be seen that the hardness and modulus of the 230 nm thick sample had considerably lower values than 147 or 400 nm films. A logical expectation would be for the result to be positioned in between 147 and 400 nm thick samples. The detrimental effect was likely caused by the increased mosaicity and the presence of differently oriented crystallites, as shown in the XRD analysis.

The reducing effect caused by the substrate was not confirmed for the 400 nm thick film and the hardness seemed to increase perpetually. Therefore, the sample was investigated further. The second reason for a more in-depth search was an attempt to find the properties corresponding solely to the ALD Cr_2_O_3_ thin film. 

**Figure 9:**
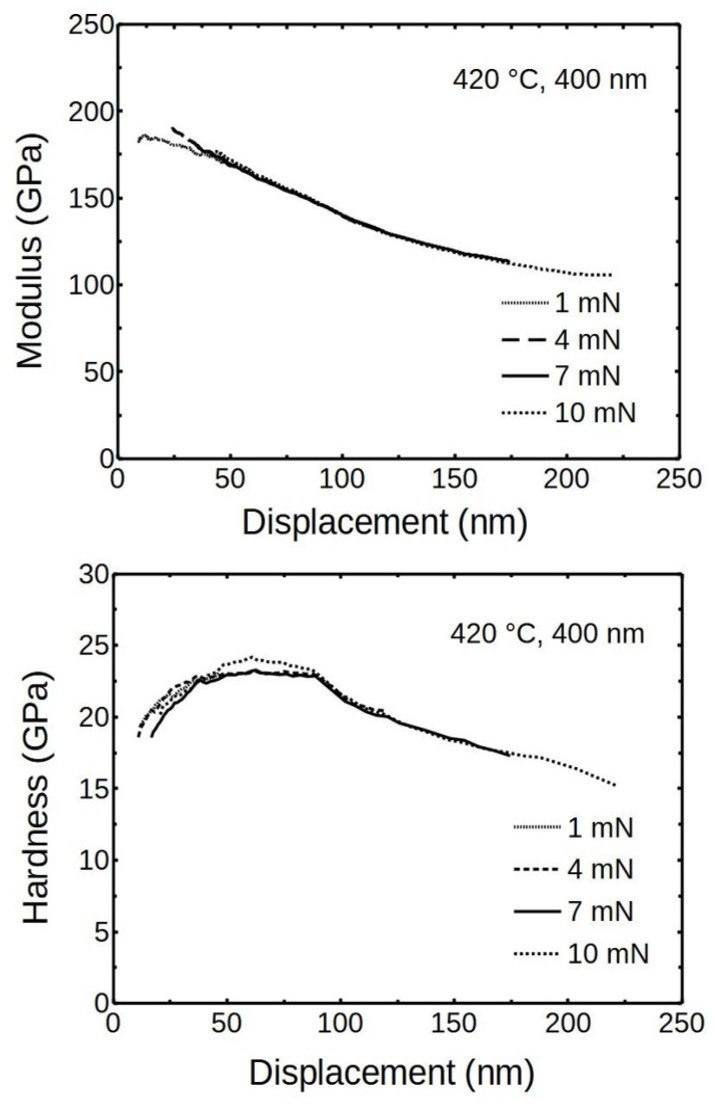
The averaged modulus and hardness of ALD chromia as measured by using different maximum forces.

The triboindenter was recalibrated to a displacement upper limit of about 300 nm because the indentation depths were expected to exceed the 100 nm limit of initial calibration. The using of maximum indentation forces of 4, 7 and 10 mN revealed that the results behaved at higher displacements, as expected, clearly showing the substrate influence (Figure 9). 

Figure 9 indicates that the averaged measurements point at the modulus of elasticity of ALD Cr_2_O_3_ was around 200 ± 20 GPa. This compares well with the results published by Pang and Khojier, but it is about 80 GPa less than in Saeki’s work [26,27]. Saeki et al. calculated and measured at room temperature the modulus of elasticity of α-Cr_2_O_3_ to be 286 GPa. First-principles calculations indicated the bulk modulus of Cr_2_O_3_ to be around 240 GPa at 300 K, which is quite close to current results [43].

The averaged results for hardness at different loads gave similar curves, overlapping and showing a “plateau” at the displacement range of 30–80 nm, where the hardness was around 22.5 ± 1 GPa (Figure 9). The initial increasing hardness values, seen in measurements done on 400 nm film by using 1 mN, can be viewed as just the lead to the plateau.

The averaged modulus of 200 GPa and hardness of 22.5 GPa are characteristics of the ALD Cr_2_O_3_ thin film deposited at given conditions. It had a remarkably high hardness compared to the values previously reported for oxide thin films deposited by ALD. In most cases, the reported values were below 15 GPa [44,45,46,47]. Although the hardness here did not reach the super-hardness level of 40 GPa, the 22 GPa would be a good starting point to develop super-hard ALD Cr_2_O_3_-based films [10]. 

The summarized results of XRD and nanoindentation of current work are gathered in Table 2.

It is clear from Table 2 that the sample with 2500 deposition cycles had markedly lower hardness and modulus compared to samples with 1600 and 4300 cycles. This could be attributed to the different crystallite orientations, as was seen from XRD (Figure 2). Crystallite orientation is known to have influence on material properties [12,48]. On the other hand, the determined XRD apparent crystallite sizes did not correlate well with respective hardnesses or moduli. The hardness value for 230 nm thick film was considerably lower than for neighboring samples, even though the crystallites had fairly similar sizes. The lower hardness could not be attributed to intrinsic stresses as the unit cell parameters in Table 1 did not indicate any remarkable stresses.

**Figure 10:**
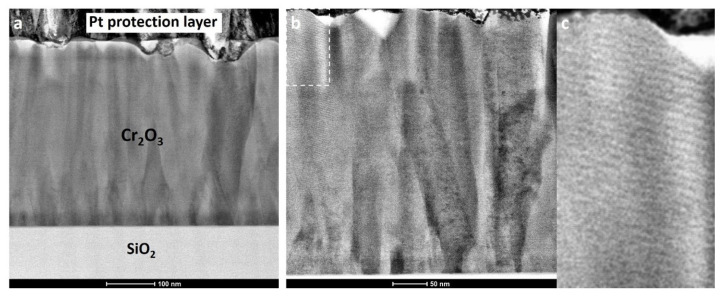
Bright-field STEM images of Cr_2_O_3_ film on SiO_2_ glass substrate deposited at 420 °C with 4300 ALD cycles. The scales are 100 nm and 50 nm for the (**a**,**b**) image, respectively. (**c**) is an enlarged cut-out of top left corner of the image (**b**).

The Cr_2_O_3_ film deposited at 420 °C with 4300 ALD cycles was also studied using scanning transmission electron microscopy. Figure 10a shows a cross-sectional bright-field STEM image of the Cr_2_O_3_ film on SiO_2_ substrate with a platinum protection layer on top. Due to the lower atomic number, the SiO_2_ substrate appears significantly brighter compared to the Cr_2_O_3_ film on the image. The image shows continuous dense Cr_2_O_3_ with no gaps or voids between various nucleated grains, which indicates a complete coalescence of the nucleation islands. The thickness of the film is relatively uniform, but some surface roughness is present. The source of the surface roughness might be a result of the formation of quite large crystallites. The thickness of the film measured on the STEM image is around 400 nm, which is in agreement with the values obtained by the XRR studies.

As can be seen by the more uniform contrast, as well as the large areas with uniform Moiré fringes (Figure 10b,c) closer to the surface of the film, the size of the grains perpendicular to the substrate tended to increase with film thickness. The increase in the lateral size of the favorably oriented grains led to the impairment of the growth of other grains and a decrease of mosaicity of the film.

Similar columnar structures were reported previously in the case of magnetron sputtered Cr_2_O_3_, but the authors did not discuss the relationship between mechanical properties and crystallite size, film thickness or the columnar microstructure [10,24]. It seems that, at the half height in the STEM image, there is a change in crystallite nucleation site count and the crystallites seem to start coalescing. This kind of growth kinetics change can introduce local mechanical stresses which, again, could initiate phase changes in a material to relieve the stress. Considering that the alpha form of Cr_2_O_3_ is a stable polymorph, the possible phase change is unlikely. On the other hand, it cannot be ruled out that residual stress somewhat changes the crystallite orientations or positioning and this could occur ex situ, over a period of time. An additional 70% thickness increase (from 230 nm to 400 nm) could, again, stabilize the structure enough that any probable changes, such as increased mosaicity, are hindered. This could be the reason why the XRD results and mechanical properties are different for Cr_2_O_3_ thin films with thicknesses of 230 and 400 nm. Unfortunately, the current set-up was to characterize the thin films ex situ and not in situ. The latter could be considered for next experiments to get more insight. Abadis et al. published a comprehensive review on stresses in thin films and related effects, including phase changes, detrimental effect on mechanical properties and so on [49].

## 4. Conclusions

The study suggests that ALD Cr_2_O_3_ is hard and relatively stiff but, in some cases, the uniform mechanical behavior might be hard to achieve. It can be concluded that the X-ray amorphous ALD Cr_2_O_3_ had the lowest dispersion of hardness and modulus, but the averaged values remained lower as compared to crystalline samples. Higher crystallinity in thin films results in higher hardness and modulus but also the higher scatter of the results, which has to be considered in applications. The optimization of deposition conditions could help to gain an ALD Cr_2_O_3_ thin film with a lower dispersion of properties.

The research showed correlation between ALD conditions, such as growth temperature, and resulting mechanical properties, such as hardness, which can be taken as a guide in application or further research. The measurements showed that the averaged hardness was 22.5 ± 1 GPa and the modulus was 200 ± 25 GPa for the film deposited at 420 °C. The hardness of thin films indicates that it could be used as starting point for developing super-hard ALD coatings. 

Atomic-layer-deposited Cr_2_O_3_ looks like a very good candidate material as a mechanically protective coating. The Cr_2_O_3_ coating would considerably enhance the surface properties of, for instance, the silicon- or silicon-oxide-based parts of micromechanical devices. Due to the conformal growth of ALD thin films, these could be applied on basically arbitrary shapes, which is very convenient in the case of micro-electromechanical systems. The actual performance, naturally, depends on the selected substrate, thin film thickness and deposition conditions (i.e., the temperature).

## Figures and Tables

**Table 1:** Summarized results of X-ray analysis of ALD Cr_2_O_3_ thin films.

Cycle Count	Thickness (nm)	Growth Temperature Tg (°C)	Crystallite Size (nm)	Integral Width β_104_	Unit Cell a (nm)	Unit Cell c (nm)
4300	400	420	34	0.46(1)	0.495	1.355
2500	230	420	37	0.49(2)	0.495	1.359
1600	147	420	30	0.52(2)	0.496	1.358
900	74	420	28.5	0.53(3)	0.495	1.359
400	28	420	18	0.61(3)		
1300	68	330	2.2			
1000	75	375	21			
900	85	465	28			

**Table 2:** XRD and averaged indentation hardness and modulus results given at 20 nm of tip displacement.

Cycle Count	Thickness (nm)	Growth Temp. T_g_ (°C)	Crystallite Size (nm)	Hardness (GPa)	Modulus (GPa)
4300	400	420	34	21.1	183
2500	230	420	37	15.9	108
1600	147	420	30	21.2	145
900	74	420	28.5	15.5	106
400	28	420	18	11.7	83
1300	68	330	2.2	12.5	105
1000	75	375	21	14.9	99
900	85	465	29	14.3	106

## Data Availability

The data presented in this study are available on request from the corresponding author.
